# The organic formation of a wellness committee: A unique, student-led approach to implementing a wellness program in medical school

**DOI:** 10.1007/s40037-020-00574-4

**Published:** 2020-04-03

**Authors:** Michael R. Brunner, Daniel Peters, Mary Virginia Portera, Irtiqa Fazili, Mary McBride, Mallie Dennis, Michael J. Herr

**Affiliations:** 1 College of Medicine, University of Tennessee Health Science Center, Memphis, TN USA; 2 College of Medicine, Department of Anatomy and Neurobiology, University of Tennessee Health Science Center, Memphis, TN USA

**Keywords:** Medical student wellness, Wellness programs, Student-led programming

## Abstract

**Background:**

Medical school wellness programs are rapidly becoming a prevalent feature of medical education. The wellness component of medical education is addressed by a multitude of different approaches, but is often led by administrative faculty rather than students.

**Approach:**

The first-year medical student authors collectively established a medical school wellness committee that is entirely student-driven. The goal of the wellness committee was to organize and promote student ideas centered on six aspects of wellness.

**Evaluation:**

The formation, initial successes, and hurdles to the inception and continuation of the committee are described in a repeatable way.

**Reflection:**

This perspective provides insight into a student-led innovation that was formally accepted by faculty and administration to serve as part of the university’s overall wellness initiatives.

## Background and need for innovation

Medical students, by their very nature of having consistently achieved high success, are at ever increasing risk for maladaptive coping mechanisms [1]. Wellness programs in medical schools seek to foster a healthy, holistic lifestyle within a very stressful academic and social environment [2]. The concept of wellness programs is not entirely new: the Kirksville College of Osteopathic Medicine in Missouri designed a wellness program in 1991 [3]. Wellness programs are also not exclusively in American medical schools; McGill University in Canada recently detailed their novel implementation of a longitudinal wellness curriculum [4]. Many other wellness programs have since arisen, the most published of which has been Vanderbilt Medical School’s Wellness Program [5, 6]. Vanderbilt’s program was seemingly the initial model for other medical schools that currently implement wellness initiatives ranging from single-day events and retreats to committees, programs, and curricula [7]. A common theme shared by these programs is that they largely rely on medical school administration to drive their formation and existence [8]. The shortcoming of this reliance was recently highlighted by Gaw as he commented on the wellness revolution and brought to attention the need for wellness programs to serve the purpose of instituting wellness as a philosophy, rather than an activity [9].

Medical students at our university have access to two supportive networks run by the college of medicine administration: a combined faculty- and peer-mentorship program that uses the name MPOWER and Student Academic Support Services and Inclusion (SASSI). The MPOWER program divides first-year medical students into four houses named after locally relevant contributors to the field of medicine. Within each of the four houses, eight to nine basic science or clinical faculty mentors are each assigned to five or six first-year medical students in smaller groups. Second-, third-, and fourth-year medical students have the option to participate longitudinally and serve as peer-mentors with each faculty group. MPOWER reflects a growing trend encouraged by the Liaison Committee for Medical Education to give students access to wellness opportunities, career exploration and advice, and other benefits [10]. SASSI is available to all students and provides services and resources to enhance learning, coping, performance, and resilience. Academic and personal counseling, student advocacy, board preparation, tutoring, and test review are a few of the many available SASSI resources. These two programs function largely independently. A group of first-year medical students recognized the need for an overarching wellness component within the college of medicine and decided to independently address this need by forming a wellness committee.

## Goal of innovation

The medical student authors of this manuscript successfully formed a wellness committee in the Spring of 2018, which allows for, and encourages, any and all medical students to actively participate in wellness initiatives. The goal of this student-formed and -led wellness committee is to create an environment that fosters collaboration and promotion of wellness on campus by utilizing the organization, creativity, and dedication of medical students. The administration has been entirely supportive of the initiative, but all wellness committee meetings and activities are solely reliant on medical students’ ideas and motivation.

## Steps taken for development and implementation of innovation

The wellness committee was formed by first-year medical students who were already existing leaders in their class. These leaders included students from the Medical Student Executive Council, the Class Executive Council, Student Affairs, and the organization Aid for the Impaired Medical Student. The wellness committee was set up as a student organization on campus and a faculty advisor was recruited. The Associate Dean of Student Affairs guided these efforts through meetings with the founding students, in which a mutual understanding of the composition of the committee was developed. The university administration approved and supported the formation and operations of the committee. A small, annual fund of $2000.00 was created by the Office of Academic, Faculty, and Student Affairs to facilitate events.

The committee decided to focus on the six aspects of wellness derived from the conceptual framework of previously published wellness program manuscripts—physical, emotional, spiritual, social, professional, and intellectual. All programs and activities promoted by the wellness committee fell under one of these six aspects. A logo incorporating these six aspects as individual icons was commissioned and designed by the university marketing department. The logo and icons were used for all communication and advertisement purposes, the development of a committee website, and printed as a banner to be displayed at sponsored events. The logo brought attention to the aspects of wellness that the committee promoted and provided for rapid association of an event with the committee. In order to initially promote awareness of the committee, a biweekly ‘Wellness Wednesday’ newsletter was designed and emailed to the entire medical student body. The newsletters functioned to aggregate and promote events and resources as parts of the six aspects of wellness using the icons for simplified recognition. Additionally, faculty members were frequently cited in the newsletters for their actions on campus to improve student wellness. The wellness committee also focused on heavily advertising an ‘Inspirational Speaker Series’, which is a lecture series newly produced by the previous class executive council to promote empathy and wellness to medical students from experienced physicians in the local community.

Monthly open meetings were held in which any medical student could participate and propose wellness ideas to committee members for help with execution and promotion. Informal subcommittees of two to three students were established to empower students to take charge and lead various wellness initiatives. One subcommittee produced a photoblog highlighting stories of students and faculty, which are published alongside the newsletter and on social media. Another planned a free yoga session hosted by a professional instructor. The success of this event has led to a continuing offering of yoga sessions both on and off campus. These events highlight the ease with which medical students were able to collectively assemble and carry out their ideas to promote wellness.

After several early successes, students interested in various aspects of wellness were invited to present their ideas to the wellness committee during monthly meetings to secure support and funding if needed to implement new concepts. The first supported idea was a culinary medicine program initiative. The program invited students to prepare healthy meals for each other in hopes of promoting healthy dietary habits and building comradery. Student response and attendance with these new programs was strongly positive and plans to continue expanding programming are currently being enacted. These efforts were highlighted to other medical students at the American Association of Medical Colleges annual meeting in 2019 through poster presentations in an effort to inspire other students to innovate at their schools.

As the time approached for second-year medical students to prepare for United States Medical Licensing Examination Step 1, leaders in the first-year class were recruited with a specific focus on those who had already shown interest in the Wellness Committee and representatives from the aforementioned student organizations. Two students were assigned as a point-of-contact with the faculty advisor to allow for accountability, and standing monthly meetings were continued to ensure the promotion of the committee and progress of events. First-year students who were genuinely interested in leading the wellness committee were charged with regularly attending the committee’s monthly meetings and promoting the committee’s initiatives. When these students progress to the end of their second year, they will again identify leadership for the wellness committee for the upcoming academic year.

## Evaluation of innovation

The initiative to create a student-run wellness committee was carried out with the goal of allowing medical students a chance to promote their own wellness-centered events with the support of the committee. The wellness committee was created to augment already existing initiatives (MPOWER and SASSI) and provide a singular source for wellness information. Attempts are being made by monthly committee meetings to consolidate wellness efforts by the university, through SASSI and MPOWER, under the jurisdiction of the wellness committee in order to more efficiently utilize resources. We have found that recruiting student leaders from previously established leadership roles provided assurance that the wellness committee would be a part of a larger discussion within each of these student organizations. As each student had proven to be a leader in their class, the committee could function synergistically to fulfill common goals without a hierarchy in leadership (e.g. president, vice president). In lieu of a hierarchical system, students are encouraged to openly participate in any of the activities the committee plans to promote. This has been mostly successful and provides certain leaders with the ability to step in and out of initiatives based on their interest and availability.

One major initial hurdle to the execution of the wellness committee initiatives was communication. The assignment of a faculty advisor was necessary to ensure the continuation of the committee as students matriculate and graduate from the university. Further, assigning two students as a point of contact to the faculty advisor was crucial to ensure continued communication with the advisor while maintaining student focus on schoolwork. The faculty advisor regularly communicated with the student points of contact to make sure committee efforts were progressing. A second hurdle was gaining recognition of the committee’s efforts by the student body. With multiple efforts to promote wellness on campus, it can be difficult for students to recognize which organization is sponsoring which event. The creation of a logo for branding purposes has helped mitigate this confusion.

Lastly, we wish to be more purposeful in our planning and measuring of the effects of the committee’s efforts. Tracking the success of the wellness committee program and student interest and involvement in the committee is an ongoing effort. Institutional Review Board approval is being sought for future qualitative studies to longitudinally track student satisfaction with wellness programming, both current and new. We are currently relying solely on attendance and feedback from students who attend the monthly committee meetings. The committee would also like to include more third- and fourth-year medical students in future programming endeavors. Few, if any, medical schools have wellness initiatives available for students in their clinical years, despite clear evidence that the challenges of medical school do not abate, and potentially worsen, during the third year [11]. The logistics of aligning schedules between the pre-clinical and clinical students contributes to this difficulty. However, we believe that the programming we have established is applicable to all medical students, and we plan to encourage involvement as extensively as possible. The manner in which this wellness committee was formed is unique and promotes student involvement at all levels.

## Critical reflection

Despite increased efforts by schools across the country, the continued attention and studies on wellness demonstrate there is still a need for innovative solutions [12–14]. The innovative formation of this student-led wellness committee provides an excellent opportunity for students to take initiative to achieve wellness during their training, alongside efforts by the administration to improve overall wellness in medical schools. Elliott et al. recently demonstrated the innovative power of the medical student body and how student innovation can lead to concrete changes [15]. Our model also allows any medical student to ‘buy-in’ to the wellness initiatives by having the ability to propose new ideas that are promoted by the committee. This wellness committee is designed to function in unison with other wellness initiatives on campus to promote and potentially measure each aspect of wellness and is used as a model for the other graduate schools that are part of our university.
